# Degradation of Exopolysaccharides from Lactic Acid Bacteria by Thermal, Chemical, Enzymatic and Ultrasound Stresses

**DOI:** 10.3390/foods10020396

**Published:** 2021-02-11

**Authors:** Carsten Nachtigall, Harald Rohm, Doris Jaros

**Affiliations:** Chair of Food Engineering, Institute of Natural Materials Technology, Technische Universität Dresden, 01062 Dresden, Germany; harald.rohm@tu-dresden.de (H.R.); doris.jaros@tu-dresden.de (D.J.)

**Keywords:** degradation, dextran, exopolysaccharides, lactic acid bacteria, molecular mass, random scission model, shearing, *Streptococcus thermophilus*, ultrasound

## Abstract

During isolation, exopolysaccharides (EPS) from lactic acid bacteria are subject of thermal, chemical, enzymatic or ultrasound stress of different intensity that may affect macromolecular properties, for instance molecular mass or (intrinsic) viscosity. These parameters are, however, crucial, as they are associated with the technofunctional potential of EPS replacing commercial thickeners in nonfermented products. The aim of this study was to systematically examine treatments EPS are usually exposed to during isolation and to investigate the underlying degradation mechanisms. Solutions (1.0 g/L) of EPS from *Streptococcus thermophilus*, isolated as gently as possible, and commercial dextran were analyzed for molecular mass distributions as representative measure of molecule alterations. Generally, acid, excessive heat and ultrasonication, intensified by simultaneous application, showed EPS degradation effects. Thus, recommendations are given for isolation protocols. Ultrasonic degradation at 114 W/cm² fitted into the random chain scission model and followed third- (*S. thermophilus* EPS) or second-order kinetics (dextran). The degradation rate constant reflects the sensitivity to external stresses and was DGCC7710 EPS > DGCC7919 EPS > dextran > ST143 EPS. Due to their exceptional structural heterogeneity, the differences could not be linked to individual features. The resulting molecular mass showed good correlation (r² = 0.99) with dynamic viscosity.

## 1. Introduction

For the production of fermented dairy products, for instance, yoghurt or (fresh) cheese, lactic acid bacteria such as *Streptococcus thermophilus*, *Lactococcus lactis* or *Lactobacillus delbrueckii* ssp. *bulgaricus* are widely used as starter cultures. Associated to their growth, some strains of these species are able to produce exopolysaccharides (EPS) in situ and are thus beneficial for the texture and sensory properties of the fermented product, as EPS enhance the water binding capacity, reduce syneresis, increase the viscosity of milk gels or enhance their stability against shearing [1,2,3,4]. This can lead to savings of commercial hydrocolloids in fermented products or even clean label products.

Amongst lactic acid bacteria, large variations in EPS structure are evident. Genera such as *Streptococcus* or *Lactococcus* produce hetero EPS that largely vary concerning monosaccharide composition, anomeric conformation of the monosaccharides, glycosidic linkages, side chains and molecular mass [5,6]. Homo EPS produced by *Weissella* or *Leuconostoc*, for instance, dextran, mainly differ in their degree of branching, linkage type of the backbone and their molecular mass [3,7]. Furthermore, EPS can be distinguished concerning their location: while free EPS (fEPS) are released into the fermentation medium, capsular or cell-bound EPS (cEPS) cover the cell surface through covalent linkages. However, up to now, little is known on relationships between specific structural elements, macromolecular properties of EPS and EPS functionality in fermented products [8]. Pronounced viscosity-enhancing properties were attributed to the presence of side chains and reduced flexibility of the molecules caused by, e.g., long backbones, β instead of α bonds, and 1,4-linkages instead of 1,2 or 1,3 [9,10]. The resulting intermolecular hydrogen bonds are responsible for hydrophilic interactions between EPS molecules and, therefore, effects on shear and intrinsic viscosity [5].

For a comprehensive macromolecular and structural analysis of EPS and a deeper insight into their functionality, EPS need to be resilient against conformational and/or structural changes by the underlying isolation procedure. Single isolation steps (Table 1) might affect the final isolate and need to be selected by, e.g., the required purity [11]. Heating is used for the detachment of cEPS [12] and may increase EPS yield [11,13], but could also reduce molecular mass and intrinsic viscosity. This leads to altered antioxidant properties as was shown for polysaccharides from *Inonotus obliquus* [14]. Protein removal is usually carried out by precipitation with strong acids, but trichloroacetic acid (TCA) may reduce the molecular mass of EPS as a consequence of acid hydrolysis [13]. Furthermore, co-precipitation can reduce EPS yield [11]. Other than acids, some authors subjected EPS containing media to alkaline conditions to release cEPS or for purification purposes [15,16].

Ultrasonication, considered a green technology due to its simple, safe and environment-friendly application [21,22], can be used for releasing cEPS and, after drying, for resolvatization and targeted physical modification. Several studies point on increased solubility [23,24] and improved functionality or bioactivity [22,25] of bacterial or plant-based polysaccharides after ultrasonication. These effects were explained by reduced molecular mass and narrow molecular mass distribution accompanied by reduced viscosity [26,27,28,29,30]. However, FT-IR or NMR experiments revealed a mostly unchanged primary structure after shearing [21,31]. The amount of degradation is determined by ultrasound parameters (frequency, amplitude) and solvation parameters (e.g., structure of the polymer, concentration, solvent, temperature) [32].

Polymer degradation mostly follows first- or second-order kinetics [22,31,33]. For a correct estimation of ultrasound-induced EPS degradation, it is necessary to know about the chain scission mechanism. Two models have mainly been used to describe chain scission of polymers: (I) The random chain scission model originally proposed by Schmid et al. [34] is based on a random breakdown of covalent bonds in the polymer chain (P(x) → P(x − y) + P(y)). The rate of degradation decreases with decreasing molecular mass of the polymer. The model can be described as shown in Equation (1):

(1) $$\frac{M_{N,\infty }}{M_{N,t}}+\ln\left(1-\frac{M_{N,\infty }}{M_{N,t}}\right)=-\frac{k_{1}}{c}t\cdot {\left(\frac{M_{N,\infty }}{m}\right)}^{2}+\frac{M_{N,\infty }}{M_{N,0}}+\ln\left(1-\frac{M_{N,\infty }}{M_{N,0}}\right)$$

(II) The midpoint chain scission model follows the assumption of a continuous degradation, and chain scission occurs always at the midpoint of the polymer (P(x) → 2·P(x/2)). It can be described with the following equation [35,36]:

(2) $$\ln\left(\frac{M_{N,0}-M_{N,\infty }}{M_{N,t}-M_{N,\infty }}\right)=k_{2}\cdot M_{N,\infty }\cdot t$$

M_N,0_, M _N,t_ and M_N,∞_ represent the number average molecular mass before ultrasonication, after a certain ultrasonication time t and at t = ∞, respectively; k_1_ and k_2_ are degradation constants of the random chain scission model and the midpoint scission model, respectively; m is the molecular mass of a monomer and c is the concentration of the polymer in solution.

In summary, exposition of EPS to thermal, physical, chemical or enzymatic stress usually applied during isolation or post-isolation processing may lead to intended or undesired changes in EPS molecules, mainly in their molecular mass. This affects further macromolecular properties such as (intrinsic) viscosity and thus EPS functionality when applied in nonfermented products. Therefore, this study aims to examine the impact of different stress factors on the molecular mass of EPS as a quick-to-determine parameter of molecule alteration. To avoid any effects of fermentation medium compounds, aqueous EPS solutions were used for this purpose. Based on the results, recommendations are given for isolation protocols to obtain EPS in their native state. Ultrasonication was also applied to examine degradation kinetics and shear resistance of EPS to link these data to structural features of the EPS.

## 2. Materials and Methods

### 2.1. Materials

Dextran from *Leuconostoc* ssp. and pronase E, a protease from *Streptomyces griseus*, were purchased from Sigma-Aldrich Chemie GmbH (Steinheim, Germany). Cryocultures of *Streptococcus thermophilus* DGCC7710, DGCC7919 (Danisco Deutschland GmbH, Niebüll, Germany) and ST143 (Christian Hansen A/S, Hørsholm, Denmark) were stored at −80 °C until use.

### 2.2. Exopolysaccharide Production and Reference Isolation Procedure

EPS of all *S. thermophilus* strains were produced in bioreactor cultivations [37,38]. Briefly, 60 g/L (DGCC7710) or 100 g/L (DGCC7919; ST143) whey permeate solution (Wheyco GmbH, Altentreptow, Germany) was enriched with 10 g/L tryptone, 2 g/L ammonium sulphate, 9 g/L glucose and 34.2 g/L lactose and fermented in 5 L reactors (Applikon Biotechnology BV, Delft, The Netherlands) at 40 °C under anaerobic conditions for approx. 24 h. pH was kept constant at 6.0 by automatic titration of 10 mol/L NaOH. Cultivations were stopped at the end of the exponential growth phase, and the media were stored at −80 °C until EPS isolation.

For the reference EPS isolation procedure, all steps considered to affect molecular mass were omitted to allow a systematic investigation later in this study (see Table 1). After diluting the cultivation broth 1:2 with 9 g/L sodium chloride +0.2 g/L sodium azide, cells were separated by crossflow filtration through 0.1 µm membranes at 40 °C (Sartorius Stedim Biotech GmbH, Göttingen, Germany). For subsequent concentration and purification, 5 kDa membranes were used and EPS precipitation in the remaining diafiltration retentate was conducted by adding two volume units of cold acetone. After storage at 6 °C for 24 h, centrifugation (4 °C, 15 min, 19,000 g), and resuspension in demineralized water, the EPS were freeze-dried (Alpha 1-2, Martin Christ Gefriertrocknungsanlagen, Osterode am Harz, Germany) and subsequently stored in a desiccator until further use.

### 2.3. Stresses applied to Exopolysaccharide Solutions

EPS solutions (1.0 g/L) were prepared by dissolving freeze-dried EPS in deionized water and gentle stirring for 24 h at room temperature. The EPS solutions were then subjected to different treatments: (a) 1 mL was incubated at 60, 80 or 90 °C for 10, 30 or 60 min each and subsequently cooled to room temperature in an ice bath; (b) 140 µL of TCA (800 g/L) was added to 1 mL EPS solution (resulting TCA concentration: 98 g/L), stirred for 30 s, incubated for 4 h at room temperature, neutralized with 1 mol/L NaOH (optional) and dialyzed against deionized water for 48 h (molecular mass cut-off: 6–8 kDa); (c) 1 mL of 2 mol/L NaOH was added to 1 mL EPS solution, incubated for 4 h or 24 h at room temperature with gentle stirring, neutralized with 1 mol/L HCl and dialyzed as described above; (d) 50 µL of a freshly prepared pronase E solution (4.8 mg/mL, pH 7.5) was added to 1 mL EPS solution adjusted to pH 7.0–8.0, incubated in a shaking water bath for 24 h at 37 °C and centrifuged for 5 min at 14,500 g and room temperature. The supernatant was dialyzed for 48 h as described above; (e) 0.5 mL of a 0.2 mol/L HCl was added to 1 mL EPS solution for pH adjustment. Subsequently, 0.25 mL of a freshly prepared pepsin solution (4 g/L) was added, the mixture incubated in a shaking water bath for 24 h at 37 °C and centrifuged for 5 min at 14,500 g and room temperature. The supernatant was dialyzed for 48 h as described above; (f) 2 mL of EPS solution was sonicated in an ultrasonic water bath (Elma Schmidbauer GmbH, Singen, Germany) at 37 kHz for 30 or 60 min at room temperature or 80 °C; (g) 2 mL EPS solution was treated with an UDS751 ultrasonic disintegrator (Topas GmbH, Dresden, Germany) at 24 kHz for 0–30 min and amplitudes from 10–100%. During treatment, the samples were cooled in an ice bath.

All experiments were carried out in duplicate or triplicate.

### 2.4. Determination of Ultrasound Intensity

The energy input of the ultrasonic disintegrator was determined calorimetrically [39,40]. The temperature of a defined amount of deionized water (m = 100.0 g) was adjusted to 20.0 ± 0.1 °C. Subsequently, sonication was performed in a thermally insulated vessel for 15 min with amplitudes adjusted to 10, 30, 40, 50, 70 and 100%. The time-dependent temperature increase of the sample was recorded with a testo 175 data logger (Testo SE & Co. KGaA, Titisee-Neustadt, Germany; accuracy: 0.1 K). From the slope dT/dt [K/s], the ultrasonic power input P [W] can be calculated as

(3) $$P=m\cdot c_{p}\cdot \frac{\mathrm{dT}}{\mathrm{dt}}$$

with c_p_ = 4.18 kJ/(kg·K) being the heat capacity of water. Ultrasound power density I [W/cm²] is defined as the power per area of the sonicator probe tip A [cm²]:

(4) $$I=\frac{P}{A}$$

In our experiments, a probe tip with a radius of 1.6 mm was used, and a linear correlation (r² = 0.99) was observed between the amplitude adjusted at the ultrasonic disintegrator and the power input (0.8 W ≤ P ≤ 9.1 W), resulting in an ultrasound density ranging from 10.0 to 114.2 W/cm².

### 2.5. Molecular Mass and Mono-/Disaccharide Determination

The molecular mass distribution of the exopolysaccharides, relative to pullulan standards, was determined with GPC-RI (Knauer Wissenschaftliche Geräte GmbH, Berlin, Germany) and described by the weight average molecular mass M_M_ [Da] and the number average molecular mass M_N_ [Da] (for details, see [38]). The polydispersity index is given by Đ = M_M_/M_N_ [-].

The mono- and disaccharide content of EPS solutions was analyzed with HPLC-RI as described previously [38]. Detection limits were 117 mg/L (glucose), 41 mg/L (galactose) and 20 mg/L (rhamnose).

### 2.6. Viscosity of Exopolysaccharide Solutions

The dynamic viscosity η [mPa·s] of aqueous exopolysaccharide solutions (assumption: ρ = 1.0 g/mL) was determined with a LOVIS rolling ball viscometer (Anton Paar GmbH, Ostfildern, Germany) [37]. The rolling time of a gold-coated steel ball (d = 1.50 mm, ρ = 7.88 g/mL) in a 1.59 mm diameter capillary was measured at 20 °C in six-fold replication at an angle of 70°.

### 2.7. Statistics

Data are expressed as arithmetic mean ± standard deviation (*n* > 2) or arithmetic mean ± half deviation range (*n* = 2). Differences were statistically tested with the Student–Newman–Keuls test (*p* < 0.05) after performing one-way analysis of variance (ANOVA) using SAS^®^ University Edition 6p.2 (SAS^®^ Institute, Cary, NC, USA).

## 3. Results and Discussion

### 3.1. Effects of Thermal Treatment on Molecular Mass

**Figure 1:**
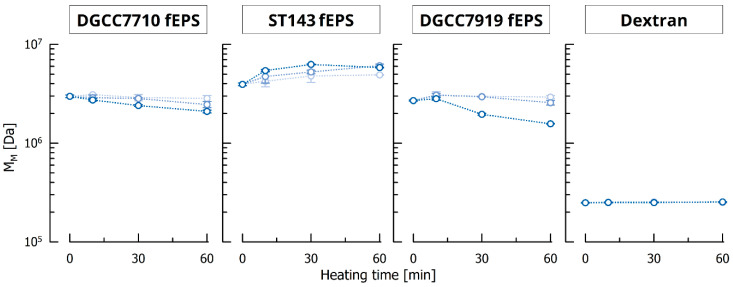
Weight average molecular mass M_M_ of EPS after thermal treatment of 1.0 g/L solutions at 60, 80 and 90 °C (light to dark blue).

The aim of a heat treatment during isolation is to inactivate cells, detach capsular EPS or improve EPS solubility [17,20]. The fEPS from the three *S. thermophilus* strains showed similar M_M_ when untreated (2.98 × 10^6^, 3.94 × 10^6^ and 2.20 × 10^6^ Da for DGCC7710 fEPS, ST143 fEPS and DGCC7919 fEPS, respectively), and M_M_ of dextran from *L. mesenteroides* was approx. one magnitude lower (2.29 × 10^5^ Da). These M_M_ were not significantly affected by thermal treatment at 60 or 80 °C for up to 60 min (Figure 1). At 90 °C, 10 min residence time did not affect M_M_ of fEPS from DGCC7710 and DGCC7919 but after 30 min, M_M_ decreased significantly to 2.40 × 10^6^ and 1.96 × 10^6^ Da, respectively, and after 60 min, to 2.10 × 10^6^ and 1.57 × 10^6^ Da, respectively. For ST143 fEPS, however, M_M_ was larger after heating, e.g., 6.27 × 10^6^ Da after 30 min at 90 °C. We assume that these EPS were not completely dissolved after stirring for 24 h at room temperature, and EPS agglomerates impaired filtration prior to GPC measurements. Improved EPS solubility and disaggregation of larger agglomerates by heating allowed the molecules to pass the filter, resulting in higher M_M_ as well as Đ (from initially 3.08 to 3.97 after 60 °C for 60 min, to 4.52 after 80 °C for 10 min, and to 4.22 after 90 °C for 10 min). This effect was overlaid by a heat-triggered molecule breakdown which was accelerated at higher temperature and longer incubation time and in accordance with findings from other authors: A decreased M_M_ and lower apparent viscosity after a heat treatment was observed for scleroglucan [41], polysaccharides from black garlic [42] and polysaccharides from *Inonotus obliquus* [14].

M_M_ of dextran was not affected by heating at all. In all sample solutions, neither mono- nor disaccharides were detected. This suggests that bond cleavage occurred anywhere within the EPS molecules and not at the end of the molecule chain.

### 3.2. Effects of Chemical Treatment on Molecular Mass

Solutions of DGCC7710 fEPS, ST143 fEPS and dextran were kept at 98 g/L TCA for 4 h, which is the time a sample is usually exposed to acidic conditions for precipitating protein and peptide residues from the fermentation medium during EPS isolation. The treatment had a significant effect on M_M_ of DGCC7710 fEPS (decrease by approx. one magnitude), but not on M_M_ of ST143 fEPS and dextran (#I in Table 2). For DGCC7710 fEPS, this pronounced decrease in M_M_ could be attenuated by introducing a subsequent neutralization step (#II), resulting in a M_M_ of 2.54 × 10^6^ Da. As some studies used also alkaline conditions for cEPS detachment and purification purposes, this was also investigated in this study. After 4 and 24 h of exposure to pH ≈ 14 with NaOH, M_M_ of DGCC7710 fEPS was reduced to 2.36 × 10^6^ and 1.82 × 10^6^ Da, respectively. The reduction was less pronounced for ST143 fEPS (2.61 × 10^6^ and 2.20 × 10^6^ Da, respectively). Molecular mass of dextran was not affected by pH ≈ 14.

For EPS isolation, it is also common practice to combine different steps (e.g., heat and acid treatment). In all subsequent experiments, we combined heating for 10 min at 90 °C, the most intense conditions where M_M_ was not affected, and subsequent cooling to room temperature in an ice bath with a TCA treatment. When the TCA treatment was applied first, this resulted, however, in a complete structure disruption (#III in Table 2): fEPS from DGCC7710 and ST143 were no longer detectable, and M_M_ of dextran was reduced to 4 × 10^4^ Da. The same effects were observed when the samples were neutralized after heating (#IV). As the samples need to be dialyzed prior to GPC, it remains unknown whether fEPS were hydrolyzed completely to monosaccharides. To avoid structure disruption, neutralization after TCA treatment prior to heating (omitting simultaneous impact of acid and heat) seems to be appropriate (#V): M_M_ did not differ significantly from untreated samples for ST143 fEPS and dextran, and M_M_ of DGCC7710 fEPS was only slightly lower (2.49 × 10^6^ Da) than that of the untreated sample. Neutralization had the same effect when the order of thermal (first) and acid (second) treatments was changed (#VI, #VII).

### 3.3. Effects of Enzymatic Treatment on Molecular Mass

The use of pronase E (optimal pH: 7.5) for hydrolysis of protein impurities did not affect M_M_ of all EPS and is therefore suitable for protein removal. Pepsin, however, reduced M_M_ significantly (DGCC7710 fEPS: 0.67 × 10^6^ Da; ST143 fEPS: 2.47 × 10^6^ Da), as the optimal pH for pepsin is 2.0. Again, dextran was not affected.

### 3.4. Effects of Ultrasonication on Molecular Mass and Viscosity

Sonication for 60 min at room temperature in an ultrasonic water bath did not cause any significant decrease of M_M_ for all EPS and can therefore be regarded as a useful tool to improve EPS solubility without molecular damage. However, when ultrasonication was performed at 80 °C for 60 min, a decrease of M_M_ to 1.28 × 10^6^ Da (DGCC7710 fEPS) and to 1.64 × 10^6^ Da (ST143 fEPS) was observed. M_M_ of dextran was not affected.

**Figure 2:**
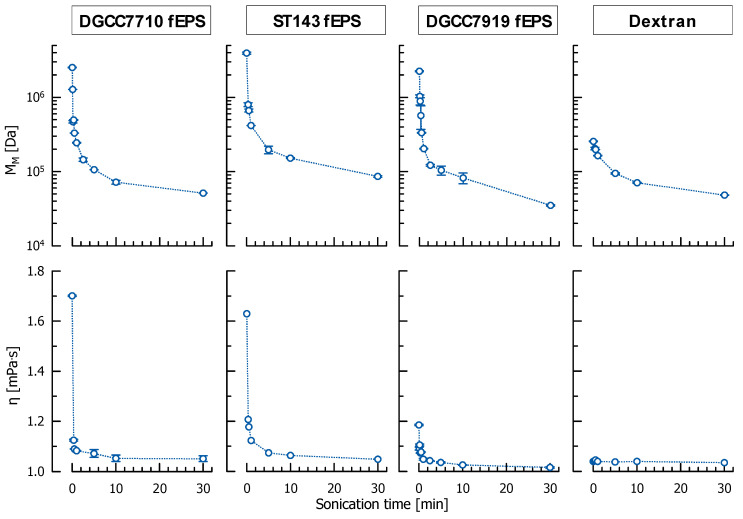
Weight average molecular mass M_M_ of EPS and dynamic viscosity η of 1.0 g/L EPS solutions after sonication with an ultrasonic disintegrator (power density: 114 W/cm²).

Sonication with the disintegrator at a power density of 114 W/cm² led to a pronounced decrease in molecular mass (Figure 2). After 20 s ultrasonication, M_M_ was already reduced to 0.50 × 10^6^ Da (DGCC7710 fEPS), 0.80 × 10^6^ Da (ST143 fEPS), 0.57 × 10^6^ Da (DGCC7919 fEPS) and 0.20 × 10^6^ Da (dextran). With ongoing sonication, the M_M_ decrease was attenuated, and M_M_ approached a limiting molecular mass M_M,∞_. DGCC7710 fEPS and dextran reached M_M,∞_ after 30 min (5.12 × 10^4^ Da and 4.82 × 10^4^ Da, respectively), i.e., no significant differences to ultrasonication for 180 min were observed. For ST143 fEPS and DGCC7919 fEPS, M_M,∞_ was reached after 60 min and was 8.59 × 10^4^ Da and 3.50 × 10^4^ Da, respectively. The molecular mass reduction also affected the viscosity of the EPS solutions. For DGCC7710 fEPS and ST143 fEPS, 5 min ultrasonication resulted in a decrease of η from 1.70 to 1.07 mPa∙s or from 1.63 to 1.07 mPa∙s, respectively (Figure 2). With further ultrasonication, the viscosity enhancing effects of EPS diminished, and η approached 1.0 mPa∙s, the viscosity of water. This effect was also observed for DGCC7919 fEPS, but less pronounced (reduction from 1.19 to 1.04 mPa∙s after 5 min) as these EPS were classified as non-ropy [38]. Dextran is a homo polysaccharide with a lower molecular mass. This resulted in a low viscosity (η = 1.04 mPa∙s) which was not affected significantly by ultrasonication.

The pronounced decrease in M_M_ by ultrasonication was already observed for other sonicated polysaccharides [35] and also for EPS treated mechanically using other systems (e.g., microfluidizer, cell disruption system) [37]. The rapid growth and collapse of cavitation bubbles, accompanied by the formation of microturbulences, is believed to be responsible for the breakage of glycosidic bonds [32]. As this occurs mainly in the center of gravity of the molecule, no monomers were formed during ultrasonication [43], as was also observed in our study. An energy density similar to our current study was applied to other polysaccharides, e.g., konjak glucomannan (24–50 W/cm²) [26], chitosan (31–62 W/cm²) [36] or polysaccharide–protein complexes from a medical fungus (10–30 W/cm²) [39], but also higher I was introduced, e.g., for schizophyllan (133–796 W/cm²) [22] or polysaccharides from *Phellinus linteus* mycelia (151–453 W/cm²) [44]. The resulting molecular mass distributions are, however, difficult to compare due to different polysaccharide structure, concentration and temperature during ultrasonication and the initial M_M_.

Recently, we demonstrated a linear dependency of η on M_M_ for DGCC7710 fEPS that were treated with different mechanical shearing systems (microfluidizer, cell disruption system) [37]. The regression function (η = 2.22 × 10^−7^ mPa∙s/Da × M_M_ + 1.03 mPa·s) was confirmed by the ultrasonication data in this current study (r² = 0.99).

**Figure 3:**
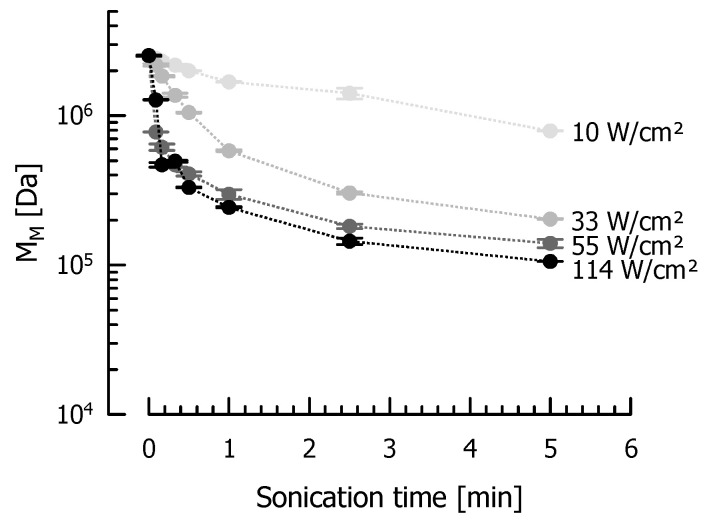
Weight average molecular mass M_M_ [Da] of DGCC7710 fEPS after sonication of aqueous solutions (1.0 g/L) with an ultrasonic disintegrator at different power densities.

DGCC7710 fEPS were also sonicated at amplitudes <100% to generate lower power inputs and thus EPS that are less affected by ultrasonication. An exponential decrease of M_M_ was observed for all investigated power densities (Figure 3). Generally, a higher I caused a more pronounced decrease of M_M_ and a lower M_M__∞_. After 5 min, M_M_ was reduced to 1.05 × 10^5^ Da (114 W/cm²), 1.40 × 10^5^ Da (55 W/cm²), 2.04 × 10^5^ Da (33 W/cm²) or 7.92 × 10^5^ Da (10 W/cm²).

**Figure 4:**
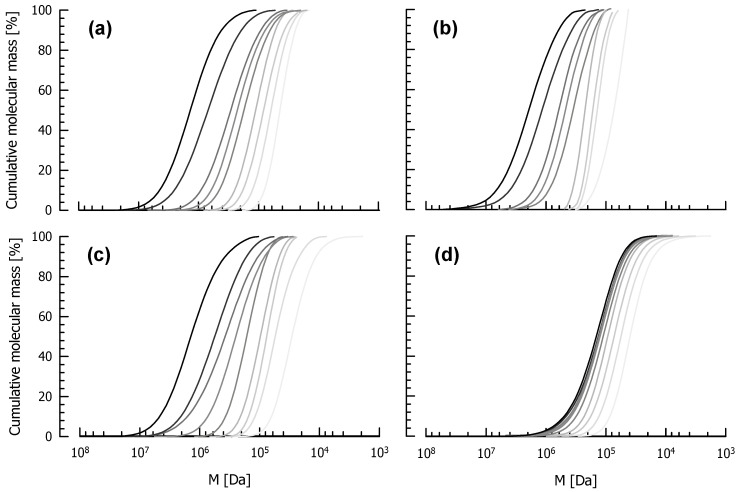
Cumulative molecular mass distributions of DGCC7710 fEPS (**a**), ST143 fEPS (**b**), DGCC7919 fEPS (**c**) and dextran (**d**). EPS solutions (1.0 g/L) were treated with an ultrasonic disintegrator (power density: 114 W/cm²) for 0 s, 5 s, 10 s, 20 s, 30 s, 2.5 min, 5 min, 10 min, 30 min (dark to light grey). M: molecular mass.

The entire functions of the cumulative molecular mass distribution of untreated and ultrasonicated EPS are shown in Figure 4. As it is the case for all biological macromolecules, untreated EPS show a relatively flat distribution (left function in each chart) and therefore a broad polydispersity that can be expressed by Đ. With increasing ultrasonication time up to 30 min, the slope of the functions increased, and polydispersity decreased from Đ = 2.06 to Đ = 1.22 (DGCC7710 fEPS), from Đ = 2.20 to Đ = 1.26 (ST143 fEPS), from Đ = 2.12 to Đ = 1.55 (DGCC7919 fEPS) and from Đ = 2.57 to Đ = 1.58 (dextran). This also corresponds to previous results obtained by other treatments [37]. Because Đ is defined as the quotient of M_M_/M_N_ and M_M_ represents larger molecules and M_N_ smaller ones in a distribution curve, a decreasing Đ indicates a faster decrease of M_M_ than M_N_, and consequently that larger molecules are more vulnerable to sonication-induced degradation than smaller ones.

### 3.5. Exopolysaccharide Degradation Kinetics

Different kinetic models were used to describe EPS degradation as a function of experimental parameters. First order degradation follows Equation (5) [22,44]:

(5) $$\ln{\;M}_{M,t}=k\prime \cdot t+{\ln\;M}_{M,0}$$

The Malhotra model follows second-order kinetics [45]:

(6) $$\frac{1}{M_{M,t}}=k\prime \prime \cdot t+\frac{1}{M_{M,0}}$$

Wu et al. [36] proposed a third-order equation to follow sonication-induced degradation of chitosan:

(7) $$\frac{1}{M_{M,t}^{2}}=k\prime \prime \prime \cdot t+\frac{1}{M_{M,0}^{2}}$$

M_M,0_ [Da] and M_M,t_ [Da] refer to the weight average molecular mass of untreated EPS and EPS after a certain ultrasonication time t [min], respectively, and k′ [1/min], k′′ [1/(Da∙min)] and k′′′ [1/(Da^2^∙min)] to the rate constants for first-, second- and third-order degradation, respectively, under defined ultrasonication conditions (sonotrode geometry, amplitude, frequency, temperature, polymer, volume and concentration of sample solution). The effect of ultrasonication on a polymer is thus reflected by k: a lower k refers to increased resistance and therefore a lower decrease of M_M_.

The weight average molecular mass of ultrasonicated EPS was fitted into the outlined functions (Table 3). None of the EPS followed first-order degradation kinetics. At the highest power density of the disintegrator (114 W/cm²), DGCC7710 fEPS, ST143 fEPS and DGCC7919 followed third-order kinetics, as indicated by the highest r². This was proposed by Wu et al. [36] for the degradation of chitosan. At lower power density, the rate constants decreased and were almost similar for second- and third-order (e.g., 0.99 for DGCC7710 fEPS at I = 10 W/cm²). This is also true for dextran (r² = 0.98 for both second- and third-order at I = 114 W/cm²). Pu et al. [27] and Lorimer et al. [46] also observed that degradation of dextran with different molecular mass distribution fitted into the second-order Malhotra model. However, EPS degradation is not a direct function of power density. Therefore, we assume that the degradation rate reaches a certain limiting value with increasing I.

Wu et al. [36] showed that k is independent from M_M,0_. Therefore, EPS with different initial molecular mass can be compared regarding their shearing resistance by means of the rate constant k′′′ at I = 114 W/cm². DGCC7710 fEPS were most vulnerable to ultrasonication, followed by DGCC7919 and dextran. ST143 fEPS showed the highest resistance against sonication as its k′′′ was lowest. With decreasing ultrasound density, k′′′ decreased, as indicated by the data for DGCC7710 fEPS. It still remains unclear whether the different behavior of dextran is due to the structural differences compared to EPS from *S. thermophilus*.

**Figure 5:**
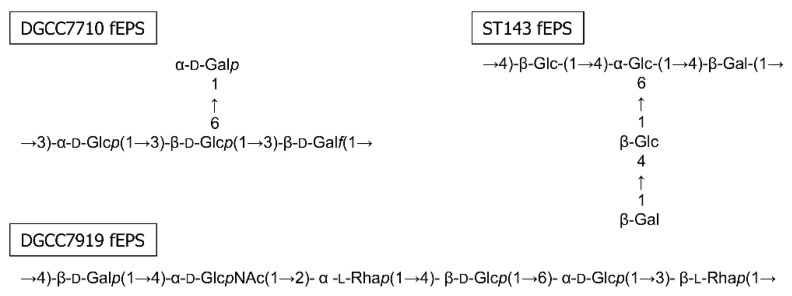
EPS structures of DGCC7710 fEPS [37], ST143 fEPS [49] and DGCC7919 fEPS [38].

Overall, our experiments showed that the EPS investigated in this study reacted differently to the thermal, chemical, enzymatic and ultrasound treatment. A higher sensitivity to chemical and thermal stress was in accordance with the observed sensitivity to mechanical energy input created by ultrasonication and was in the order DGCC7710 fEPS > DGCC7919 fEPS > dextran > ST143 fEPS. The EPS differ strongly in their chemical structures (Figure 5). Other than a different monosaccharide composition (glucose, galactose, rhamnose), these were differently linked. In the EPS backbone, 1,2- and 1,3-glycosidic linkages (as found solely in DGCC7710 fEPS) lead to more flexible molecules than 1,4-linkages (as found solely in ST143 fEPS) [5,47,48]. Enhanced flexibility is supposed to be associated with reduced sensitivity to mechanical energy input. Furthermore, β-linked monomers (as found mainly in ST143 fEPS) showed reduced flexibility compared to α-linkages (50% in DGCC7710 fEPS and DGCC7919 fEPS, 20% in ST143 fEPS) [10]. Tuinier et al. [9] observed that the presence of side chains impeded the entanglement of polysaccharide molecules and reduced the flexibility of the EPS backbone. In our study, the EPS with the longest side chain (ST143 fEPS) was least sensitive to external stress. Dextran, composed of a flexible α-1,6-linked glucopyranose chain with a low amount of 1,3,6-linkages [37], and DGCC7710 fEPS have monomeric side chains, but were differently affected by thermal, chemical and ultrasound treatments. As specific structural features interact with each other, a conclusive evaluation of the stress sensitivity induced by specific EPS structural details still remains challenging. To link distinct structural features to the sensitivity against external stresses, it is necessary to compare two EPS that differ in only one parameter, e.g., the presence of a monomeric side chain. This could be realized by enzymatic removal of those side chains of an EPS from lactic acid bacteria.

### 3.6. Investigation of Chain Scission Models

**Figure 6:**
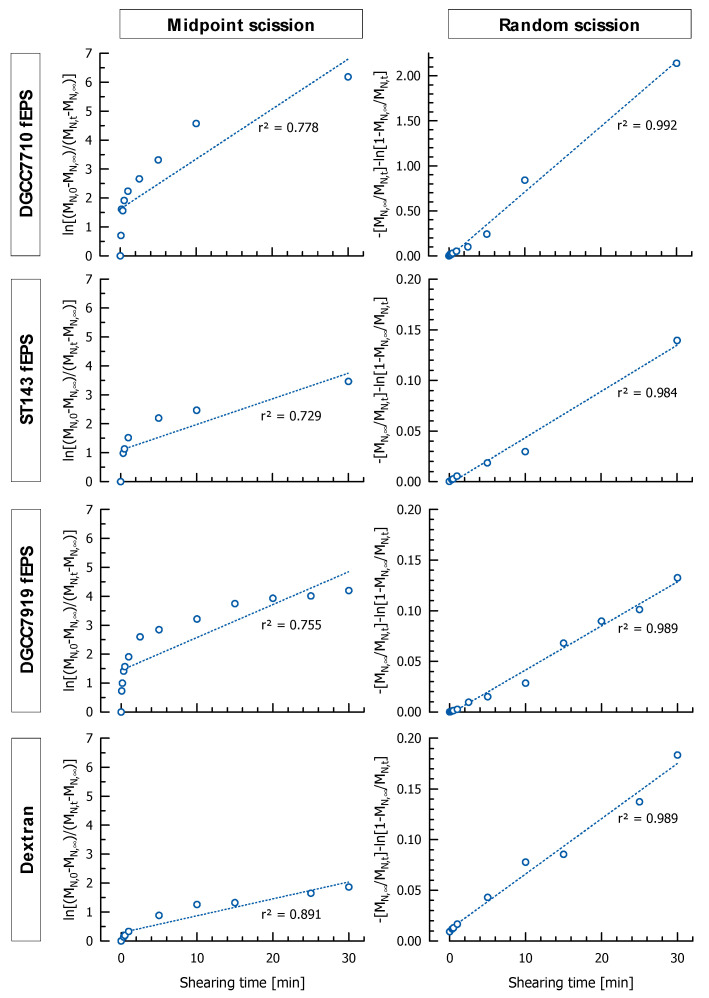
Linearization plot of the midpoint scission model (Equation (2)) and the random scission model (Equation (1)) for ultrasonicated EPS (power density: 114 W/cm²).

After linearization of Equations (1) and (2), the number average molecular mass of the EPS can be used to determine the localization of chain scission during ultrasonication. M_N,∞_ was determined experimentally by sonication with an ultrasonic disintegrator for three hours as proposed earlier by Wu et al. [36]. Data of all investigated EPS fitted closely to the random scission model (r² > 0.98) but not to the midpoint scission model (r² < 0.89), suggesting an unsystematic breakdown of glycosidic bonds with evenly distributed scission points over the entire EPS molecule during ultrasonication (Figure 6). HPLC analyses supported these findings by revealing that no mono- and disaccharides were released during ultrasonication.

The linear dependency of η on M_M_, evident for ultrasonication and shearing with a microfluidizer or a cell disruption system, indicates the same degradation mechanism of molecule breakdown for the different treatments. This is also consistent with findings from NMR experiments where we observed that microfluidization does not alter EPS structure [37]. Studies point to unaltered structures after ultrasonication, however, in some studies, the FT-IR measurements might not have been sensitive enough to detect minor changes of EPS structure [28,30,44,50,51]. The random chain scission was proved for the majority of polysaccharides, e.g., chitosan [36], konjak glucomannan [26,33], pectin [51], (1-3)(1-6)-α-D-glucans from *Leuconostoc citreum* [21] or polysaccharides from *Sargassum pallidum* [50]. For some polysaccharides, such as carboxylic curdlan [28] and six different dextrans from *L. mesenteroides* [27], however, midpoint chain scission was observed, which is in contrast to our study, where dextran also fitted the random chain scission model. This may be due to the fact that other authors used the viscosity-average molecular mass (instead of number-average) or did not state which molecular mass was used for the calculations. Furthermore, the degree of branching may also affect the degradation mechanism of dextran.

## 4. Conclusions

The investigated EPS were largely affected by acid and ultrasonication treatment. As the M_M_ decrease was only 8% at maximum after 10 min at 90 °C, we suggest this step for cEPS detachment or resolvatization of isolated EPS. Furthermore, applying combinations of heat with other treatments simultaneously should be avoided, as this enhanced the degrading effect of ultrasound or pH (used for acid protein precipitation, leading to acid EPS hydrolysis). EPS degradation can be further reduced by replacing acid precipitation by enzymatic hydrolysis for protein removal. The ultrasonic disintegrator was shown to be inappropriate for EPS isolation because it rapidly reduced M_M_ of all the exopolysaccharides. On the other hand, it may be useful for a targeted modification of EPS, especially at lower power density, and for the investigation of degradation mechanisms and kinetics. We observed that all EPS chains were broken randomly (Schmid model) following third- (DGCC7710 fEPS, DGCC7919 fEPS, ST143 fEPS) or second-order kinetics (dextran). The sensitivity to ultrasonication at 114 W/cm² was highest for DGCC7710 fEPS (k′′′ = 1.80 × 10^−11^), followed by DGCC7919 fEPS (2.02 × 10^−11^), dextran (3.60 × 10^−12^) and ST143 fEPS (5.15 × 10^−12^). The data confirmed a linear relationship between M_M_ and η, as recently published for other shearing systems. Ultrasonication showed to be a promising tool to easily adjust η and M_M_ of EPS by selecting the appropriate sonication parameters (amplitude, time) after empirical calibration. This allows to obtain EPS isolates with desired functionality for their use in nonfermented foods and beverages.

## Figures and Tables

**Table 1:** Processing steps commonly used for exopolysaccharides (EPS) isolation and purification ^1^.

Processing Step	Common Methods
Detachment of cEPS	Heating ^2^, ultrasound ^2^, NaOH treatment ^2^
Cell separation	Microfiltration, centrifugation
Protein removal	TCA ^3^ precipitation ^2^, enzymatic hydrolysis ^2^
Concentration	Microfiltration, ultrafiltration
Purification	Diafiltration, dialysis, precipitation, NaOH treatment ^2^, preparative size exclusion chromatography
Drying	Freeze drying, spray drying
Resolvatization	Heating ^2^, ultrasound assistance ^2^

^1^ taken from [1,13,17,18,19,20]; ^2^ effects on EPS investigated in this study; ^3^ TCA: trichloroacetic acid.

**Table 2:** Weight average molecular mass M_M_ of EPS after thermal and acid stress of 1.0 g/L solutions in different treatment order. Ref: reference; b.d.l.: below detection level. Mean values with different superscripts in a column indicate statistical differences (α = 0.05).

	Treatment Order			M_M_ [×10^6^ Da]		
	Acid Treatment ^1^	Neutralization ^2^	Heating ^3^	DGCC7710 fEPS	ST143 fEPS	Dextran
Ref	-	-	-	2.98 ± 0.09 ^a^	3.94 ± 0.12 ^a^	0.22 ± 0.01 ^a^
I	1	-	-	0.36 ± 0.02 ^c^	3.53 ± 0.13 ^a^	0.23 ± 0.01 ^a,b^
II	1	2	-	2.54 ± 0.15 ^b^	3.62 ± 0.02 ^a^	0.21 ± 0.01 ^a,b^
III	1	-	2	b.d.l.	b.d.l.	0.04 ± 0.01 ^c^
IV	1	3	2	b.d.l.	b.d.l.	0.04 ± 0.01 ^c^
V	1	2	3	2.49 ± 0.06 ^b^	3.70 ± 0.37 ^a^	0.22 ± 0.01 ^a,b^
VI	2	-	1	0.45 ± 0.01 ^c^	3.79 ± 0.18 ^a^	0.22 ± 0.01 ^b^
VII	2	3	1	2.22 ± 0.01 ^b^	3.41 ± 0.15 ^a^	0.23 ± 0.01 ^a,b^

^1^ 98 g/L trichloroacetic acid (TCA), incubation time: 4 h; ^2^ 1 mol/L NaOH; ^3^ 90 °C, 10 min and subsequent cooling to room temperature in an ice bath.

**Table 3:** Rate constants k′, k′′ and k′′′ of different degradation models, applied to ultrasonicated EPS at different power density I [W/cm²].

EPS	I [W/cm²]	1st Order Degradation		2nd Order Degradation		3rd Order Degradation	
		k′ [1/min]	r²	k′′ [1/(Da∙min)]	r²	k′′′ [1/(Da²∙min)]	r²
DGCC7710 fEPS	114	−4.76 × 10^−1^	0.59	1.74 × 10^−6^	0.93	1.80 × 10^−11^	0.99
	55	−4.13 × 10^−1^	0.58	1.34 × 10^−6^	0.92	1.19 × 10^−11^	0.99
	33	−4.96 × 10^−1^	0.83	9.36 × 10^−7^	0.98	4.59 × 10^−12^	0.98
	10	−4.03 × 10^−1^	0.94	1.86 × 10^−7^	0.99	1.66 × 10^−13^	0.99
ST143 fEPS	114	−3.75 × 10^−1^	0.64	8.51 × 10^−7^	0.94	5.15 × 10^−12^	0.99
DGCC7919 fEPS	114	−5.04 × 10^−1^	0.64	1.88 × 10^−6^	0.88	2.02 × 10^−11^	0.96
Dextran	114	−1.76 × 10^−1^	0.93	1.26 × 10^−6^	0.98	3.60 × 10^−12^	0.98

## Data Availability

The datasets generated for this study are available on request to the corresponding author.
